# Suppression of a Prolyl 4 Hydroxylase Results in Delayed Abscission of Overripe Tomato Fruits

**DOI:** 10.3389/fpls.2019.00348

**Published:** 2019-03-28

**Authors:** Andreas Perrakis, Craita E. Bita, Stilianos Arhondakis, Afrodite Krokida, Khansa Mekkaoui, Dusan Denic, Konstantinos N. Blazakis, Dimitrios Kaloudas, Panagiotis Kalaitzis

**Affiliations:** Department of Horticultural Genetics and Biotechnology, Mediterranean Agronomic Institute of Chania (MAICh), Chania, Greece

**Keywords:** tomato, prolyl-4-hydroxylase, abscission, overripe fruit, Arabinogalactan proteins, cell-wall

## Abstract

The tomato pedicel abscission zone (AZ) is considered a model system for flower and fruit abscission development, activation, and progression. O-glycosylated proteins such as the *Arabidopsis* IDA (INFLORESCENCE DEFICIENT IN ABSCISSION) peptide and Arabinogalactan proteins (AGPs) which undergo proline hydroxylation were demonstrated to participate in abscission regulation. Considering that the frequency of occurrence of proline hydroxylation might determine the structure as well the function of such proteins, the expression of a tomato prolyl 4 hydroxylase, SlP4H3 (*Solanum lycopersicum* Prolyl 4 Hydroxylase 3) was suppressed in order to investigate the physiological significance of this post-translational modification in tomato abscission. Silencing of SlP4H3 resulted in the delay of abscission progression in overripe tomato fruits 90 days after the breaker stage. The cause of this delay was attributed to the downregulation of the expression of cell wall hydrolases such as SlTAPGs (tomato abscission polygalacturonases) and cellulases as well as expansins. In addition, minor changes were observed in the mRNA levels of two SlAGPs and one extensin. Moreover, structural changes were observed in the silenced SlP4H3AZs. The fracture plane of the AZ was curved and not along a line as in wild type and there was a lack of lignin deposition in the AZs of overripe fruits 30 days after breaker. These results suggest that proline hydroxylation might play a role in the regulation of tomato pedicel abscission.

## Introduction

Abscission is the process of organ separation in plants with significant implications in crop productivity considering that fruit and flower abscission might affect yield in several crops (Addicott, 1982; Tranbarger et al., 2017). Some plant species such as *Arabidopsis* and tomato are considered model systems for abscission studies, particularly *Arabidopsis* for flower abscission and tomato for pedicel abscission.

There are several advantages to study abscission in tomato considering the high-quality annotation of the tomato genome, updated and detailed transcriptome databases such as Tomexpress^1^ and tomato functional genomics database^2^, a tomato abscission zone-specific microarray (Meir et al., 2010; Sundaresan et al., 2016), abscission mutants such as the *jointless (j)* locus which lack the abscission zone (AZ) (Mao et al., 2000), and well-established transformation and, recently, genome editing methodologies. The AZ-specific microarray was constructed after RNAseq of samples from various developmental stages of leaf and flower AZs which resulted in the identification of 8,823 transcripts (Sundaresan et al., 2016).

The tomato pedicel comprises a distinct AZ at a predetermined position which is responsible for the detachment of either the flower or the fruit when the abscission process initiates. This AZ is comprised of six to eight cell layers at the open flower stage. At the overripe fruit stage, the AZs showed lignin deposition (Iwai et al., 2013; Tsuchiya et al., 2015) while no cellulose synthase, xyloglycan endotransglycosylase/hydrolase, and expansin signals were observed (Tsuchiya et al., 2015). A group of the expansin gene family comprising 16 members showed differential expression patterns in tomato leaf and flower AZs while most of them were mainly expressed in tomato leaf AZs (Sundaresan et al., 2016). Two of them, SlEXP1 and SlEXP11 were shown to be upregulated in flower AZs after 6 h of ethylene treatment (Wang et al., 2013).

Transcriptome analysis revealed that several key regulators of meristem-associated functions such as a tomato homolog of WUSCHEL (LeWUS), GOBLET (GOB), LATERAL SUPPRESSOR (*Ls*), and Blind (*Bl*) are specifically expressed in the pedicel AZ (Nakano and Ito, 2013). Among the transcription factors which are expressed in pedicel AZ, there is a member of the ERF (ethylene response family), the SlERF52 which was reported to play a regulatory role in the induction of the cell wall hydrolases during abscission progression (Nakano et al., 2014). Downregulation of SlRF52 delayed pedicel abscission due to the lack of upregulation of tomato abscission PGs (TAPGs) and cellulases (Nakano et al., 2014).

In *Arabidopsis*, the hormone peptide IDA (inflorescence deficient in abscission) and their interacting receptor-like kinases HAESA and HAESA-like2 are considered master regulators of flower abscission (Butenko et al., 2003; Stenvik et al., 2008). The *Arabidopsis* IDA peptide is hydroxylated at a proline residue in position 7 and this hydroxylation event increased the activity of the peptide by 30-fold, indicating the functional significance of proline hydroxylation for IDA (Butenko and Simon, 2014). Five IDA genes were identified in tomato and only one of them, SlIDA1, was expressed in the leaf AZ as well as the petiole (Tucker and Yang, 2012). However, gene expression data alone could not lead to any conclusion related to the role of IDA in tomato abscission (Tucker and Yang, 2012). Moreover, there is no information whether the tomato IDA peptides are proline hydroxylated and the physiological importance of this hydroxylation. There are also 15 CLAVATE3/EMBRYO-SURROUNDING REGION (CLE)-secreted peptide genes which exhibited tissue- and organ-specific expression patterns while strong expression was observed during fruit development and ripening (Zhang et al., 2014). However, it has not been reported whether any of them is expressed in fruit or leaf AZs.

There are 10 tomato prolyl 4 hydroxylases (P4Hs) which were shown to be involved in cell division and expansion of leaves by using a virus-induced gene silencing (VIGS) approach (Fragkostefanakis et al., 2014). These phenotypes might be related to observed alterations in the protein content of substrate proteins such as Arabinogalactan proteins (AGPs) and extensins (Fragkostefanakis et al., 2012 , 2014). Moreover, the use of 2-oxoglutarate analog pyridine 2,4-dicarboxylate resulted in a decrease in hydroxyproline content and in epitope bound AGPs according to western blot analysis (Fragkostefanakis et al., 2018). In addition, three *Arabidopsis* P4Hs were reported to have a regulatory role in the root hair elongation process (Velasquez et al., 2011 , 2015). The AtP4H2, AtP4H5, and AtP4H13 T-DNA knock out mutants exhibited a short root hair phenotype supporting the model that proline hydroxylation of hydroxyproline-rich glycoproteins and particularly extensins is important for cell wall assembly and function (Velasquez et al., 2011 , 2015).

In this study, we report that suppression of SlP4H3 expression resulted in the delay of pedicel abscission in overripe tomato fruits. This delay was attributed to suppression of expression of abscission-specific cell wall hydrolases.

## Materials and Methods

### Construction of Binary Vector, Agro-Mediated Transformation and Transgenic Plants

The RNAi-SlP4H3 construct was generated by the amplification of a 838 bp fragment corresponding to base 168-1006 of the cDNA SlP4H3 gene (Solyc02g083390.2) using *the* SlP4H3 primers (Supplementary Table 1), containing *attb* sites and subcloned into the pDNR221 (accession number AF485783) entry vector (Invitrogen). The construct was recombined into the pHellsgate12 vector (a.n. AF489904) (CSIRO, Australia), using Gateway cloning technology (Invitrogen, Carlsbad, CA). The insertion in the binary vector was verified by sequencing, and used to transform tomato cotyledons Ailsa Craig (AC) by *Agrobacterium tumefaciens* (strain LBA4404) according to Fillatti et al. (1987) with some modifications. For positive control, an empty vector (pBi121, a.n. AF485783) was inserted into *A. tumefaciens* (strain LBA4404) and used for transformation of Ailsa Craig cotyledons. Transgenic plants were detected with PCR using the gene-specific primers for *nptII* cDNA. Nine independent transgenic lines as well as the empty vector (pBi121) were grown in parallel with an open hydroponic system in two separate net chambers in the experimental greenhouse of M.A.I.Ch. Tissues of AZs of overripe fruit 30 and 90 days after breaker from RNAi and wild type plants were collected, frozen in liquid nitrogen, and stored at −80°C (Alba et al., 2005).

Approximately 5 μg of genomic DNA was extracted from transgenic and wild type plants as described by Sambrook et al. (1982) with few modifications, in order to confirm construct integration into the genome. PCR-specific primers of *nptII* and SlP4H3 cDNAs were designed (Supplementary Table 1) and used to amplify a partial fragment of *nptII* and SlP4H3. In brief, for each reaction, 50 ng of template DNA, 1X buffer, 200 μM dNTPs, 1.0 μM of each primer, and 1.25 units *Taq* Polymerase (Invitrogen, ThermoFisher Scientific, Walttham, MA USA) were used in a final volume of 20 μl. Thermal conditions were 2 min at 96°C for denaturation, followed by 35 cycles of 1 min at 96°C for denaturation, 1 min at 55°C for annealing, and 2 min at 72°C for extension, followed by the last step at 72 C for 10 min. For negative control, distilled water was used. Amplification products were loaded in a 1.5% agarose gel with 1xTBE running buffer, using a 1 kb DNA ladder (GeneRuler, Fermentas Life Sciences) and stained with GelRed (Biotium).

### RNA Extraction and cDNA Synthesis

Total RNA was isolated from 200mg of tissue of Br + 30 and Br + 90 fruit AZs as well as leaf AZs, from an empty vector (pBi121) line and three SlP4H3 RNAi lines (#1, #6, and #7). Tissue was grounded in liquid nitrogen and total RNA was extracted by using the RNeasy^®^ plant mini kit (QIAGEN) and PureLink™ RNA mini kit (ThermoFisher, Walttham, MA, USA). The DNAseI-RNAse free enzyme (ThermoFisher Scientific, Walttham, MA, USA) was used to remove the genomic DNA. Approximately 1 μg of total RNA was reverse transcribed using SuperscriptII^®^ Reverse Transcriptase (ThermoFisher Scientific, Walttham, MA, USA) and cDNA synthesis was performed following manufacturer’s instructions using oligodT_12–18_ primers.

### qRT-PCR Analysis

Gene expression analysis of selected genes was performed using a 48-well StepOnePlus™ Real-Time PCR System (ThermoFisher Scientific, Walttham, MA, USA) on three independent biological replicates. cDNA samples were prepared as described above and normalized using actin-specific primers (Supplementary Table 1). Standard dilution curves were performed for each PCR amplicon, and all data were normalized at the level of SlACTIN transcript. Primers were designed using the Primer Express v2.0 software (Applied Biosystems, Foster City, Calif) (Supplementary Table 1). The qRT-PCR reaction (20 μl) mix consisted of gene-specific primers, as shown in Supplementary Table 1, SYBR™ Select Master Mix (ThermoFisher Scientific, Walttham, MA, USA), cDNA templates, and were run in StepOne™ Real-Time PCR system. The thermal cycling conditions were 50°C for 2 min, 95°C for 10 min followed by 95°C for 15 s, 60°C for 30 s, and 72°C for 30 s for 40 cycles. Data were analyzed using the 2^−ΔΔCT^ method (Livak and Schmittgen, 2001) and presented as relative levels of gene expression. Standard errors were calculated for all mean values.

### Statistical Analysis

The numerical data were analyzed and post-processed using the statistics toolbox of MATLAB (The Mathworks Inc., Natick, MA, USA). The standard descriptive statistical methods were applied on the numerical findings and the data re-expressed as mean ± standard errors (SEs). Then one-way analysis of variance (one-way ANOVA) was applied on the data of each line, to analyze and determine possible statistically significant differences in the data sets. Furthermore, we used a *post hoc* multicomparison approach (Tukey’s honest significant difference criterion, 95% confidence interval) to identify the differences between wild type (WT) and each RNAi line.

### LIGNIN Staining With Phloroglucinol-HCl

Approximately 0.3 g of phloroglucinol was dissolved in 10 ml of absolute ethanol to prepare a 3% phloroglucinol solution. One volume of concentrated HCl (37 N) was mixed with two volumes of 3% phloroglucinol in ethanol to prepare the phloroglucinol-HCl (Ph-HCl). The AZ sections were transferred to a 2.0-ml microcentrifuge tube and 1 ml of Ph-HCl solution was added to the tube. The tube was shaken gently to assure that all the sections are stained. The observation was made under bright-field lighting microscope.

### Plant Material and Flower Abscission Zone Sections

The tomato plants were grown in the greenhouse of the Mediterranean Agronomic Institute of Chania under regular conditions. The leaf abscission explants were prepared from stems of mature tomato plants comprising around 10 cm of stem section with the petiole after removing the leaf blade. The explants were then placed upright in beakers with water and treated with approximately 15 μl L^−1^ of ethylene in jars with an open system installation for 48 h. The flower AZs were fixed in 10% formalin, then dehydrated through a series of graded ethanol and embedded in paraffin by using a paraffin embedding station (Leica TP1020). The AZs were then used to prepare longitudinal sections with a rotary microtome (Leica RM2135) for image analysis with a stereomicroscope Leica MZ7.5 (Meyer Instruments Inc.), a camera ProgRes^®^ C12 plus (Jenoptik), and the CapturePro^®^ 2.1 Image Acquisition Software.

## Results

### Suppression of SlP4H3 by Using an RNAi Approach

Ten P4H genes were identified in the *S. lycopersicum* genome while a dendrogram which was prepared after comparison of their deduced amino acid sequences indicated clustering into three groups (Fragkostefanakis et al., 2014). Among them, 3 tomato P4Hs, SlP4H3, SlP4H8, and SlP4H10, and another 6 out of 13 *Arabidopsis* P4Hs were grouped into the same subcluster of cluster A (Fragkostefanakis et al., 2014). This indicates higher homology of their deduced amino acid sequence to *Arabidopsis* P4Hs compared to the other tomato P4Hs. *Arabidopsis* P4Hs were reported to be involved with a regulatory role in growth programs such as root hair elongation (Velasquez et al., 2011, 2015). Moreover, silencing of additional tomato P4Hs by using stable transformation and transient approaches such as VIGS indicated that SlP4H3 exhibited interesting phenotypes compared to other P4Hs (data not shown). Therefore, among the three, the tomato SlP4H3 was initially selected for further investigation.

**Figure 1:**
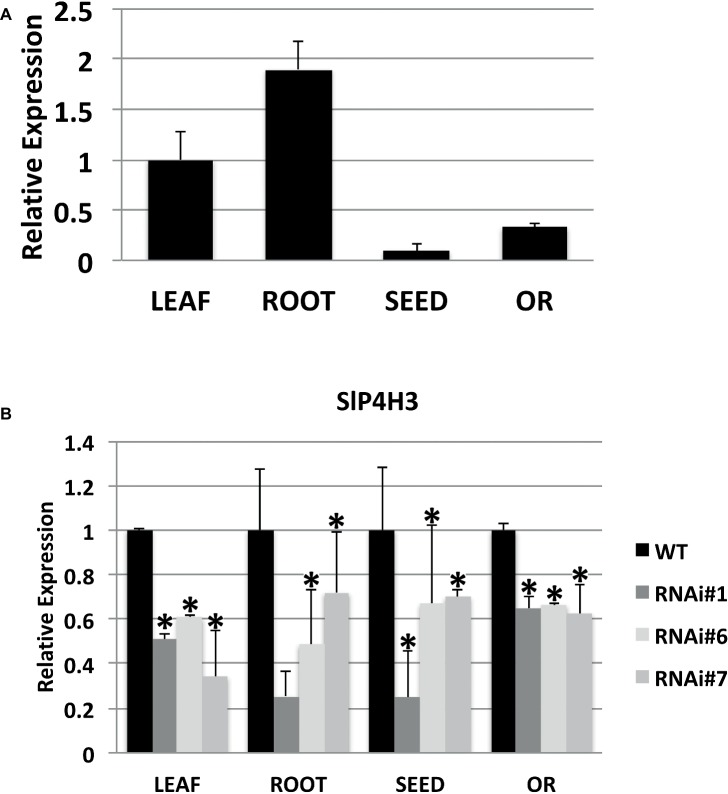
Expression of the SlP4H3 in leaf, root, seed, and overripe fruit of wild type and P4H3 RNAi lines #1, #6, and #7. **(A)** Relative expression of SlP4H3 in leaf, root, seed, and overripe fruit. **(B)** Relative expression of SlP4H3 in similar tissues of P4H3 RNAi lines #1, #6, and #7 compared to the wild type. The relative expression was calculated according to the comparative Ct method by using actin as internal standard. The asterisk indicates statistically significant differences.

A specific RNAi silencing construct for SlP4H3 whose expression was directed by the 35S promoter was transformed into wild type (*Ailsa Craig*) tomato plants by using *A. tumefaciens*-mediated transformation and nine independent transformants were generated. The construct integration was confirmed by the presence of *nptII* in each line and three lines, RNAi#1, RNAi#6, and RNAi#7, were further characterized at the T2 generation. The expression of SlP4H3 was determined at various tissues such as leaf, root, seed, and overripe fruit by qPCR (Figure 1A). The higher expression was determined in root and leaf while lower levels of expression were determined in the seed and overripe fruit (Figure 1A). Moreover, the levels of suppression of SlP4H3 were determined in the same tissues in all three lines, RNAi#1, RNAi#6, and RNAi#7 (Figure 1B). Significant suppression was observed in all tissues with the highest decrease in expression detected in leaf tissue among the three RNAi lines (Figure 1B). Moreover, the SlP4H3 RNAi line #1 showed the highest percentage of suppression in root and seed tissue (Figure 1B).

### SlP4H3 RNAi Lines Exhibited a Delayed Abscission Phenotype

**Figure 2:**
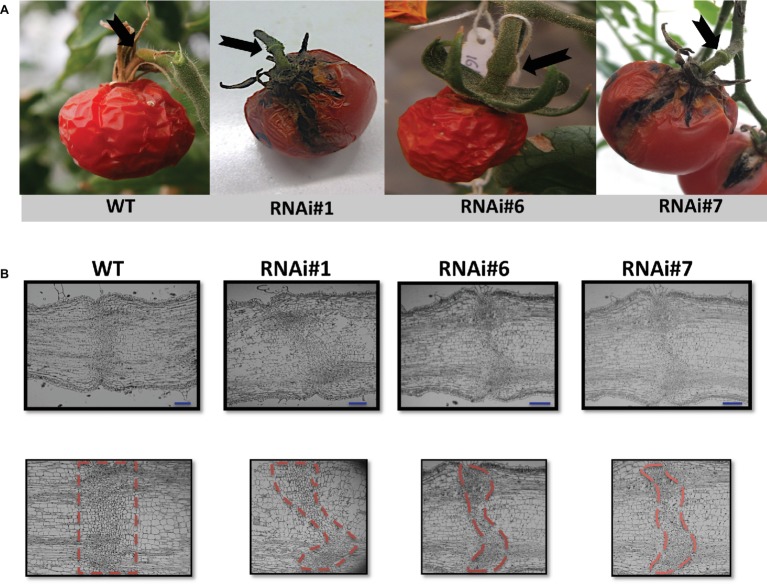
Delay of overripe fruit abscission and morphological structure of AZs in SlP4H3 RNAi lines. **(A)** Final stage of abscission of a wild type overripe fruit 90 days after breaker. Representative AZs of overripe fruits 90 days after breaker in the three SlP4H3 RNAi lines #1, #6, and #7. The arrows point to the AZ. **(B)** Longitudinal sections of petiole AZs at the stage of open flower in the SlP4H3 RNAi lines and wild type. The dotted-line contour defines the abscission zone based on the visible separation layer. The blue line represents 1 mm.

Wild type overripe tomato fruits abscised naturally usually around 90 days after the breaker stage under normal growing conditions (Figure 2A). However, the overripe fruits of three SlP4H3 RNAi lines did not abscise 90 days after breaker as shown in Figure 2A. Even tomato fruits, which were in terms of development at the final stages of senescence, showed a delayed abscission phenotype (Figure 2A). This significant delay in fruit abscission was observed in all nine independent transformants. Moreover, the hydroxylation of specific proline residues is important for IDA activity, suggesting a putative role for this post-translational modification in abscission regulation in *Arabidopsis* (Butenko and Simon, 2014).

**Figure 3:**
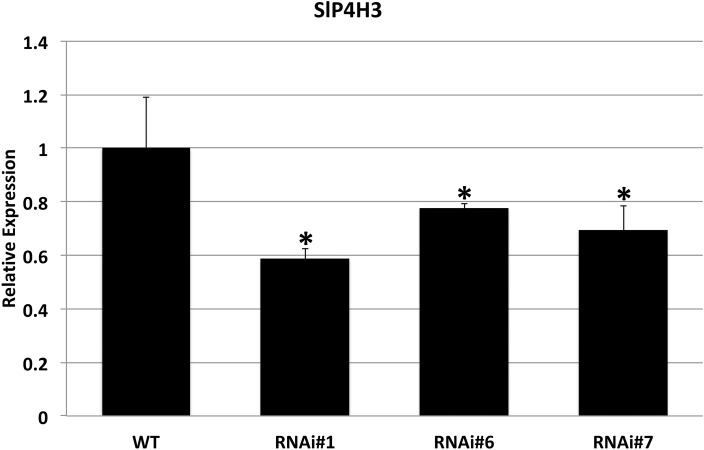
Expression analysis of the SlP4H3 in the AZs of overripe fruits 90 days after breaker in the three SlP4H3 RNAi lines #1, #6, and #7 and wild type (WT) plants. Relative expression of SlP4H3 transcript in three RNA lines and WT AZs. The relative expression was calculated according to the comparative Ct method by using actin as internal standard. The asterisk indicates statistically significant differences.

The specific repression of SlP4H3 in the fruit AZs of wild type and RNAi lines was verified by determining its expression levels. Total RNA was extracted from AZs of wild type and RNAi lines #1, #6, and #7 fruits 90 days after the breaker stage and used for RT-qPCR analysis (Figure 3). AZs of overripe fruits were used for SlP4H3 expression by using qPCR indicating that the expression levels were partially suppressed in all three lines compared to the wild type (Figure 3). The expression of the additional tomato P4Hs was investigated in the fruit AZ 90 days after the breaker stage in order to determine whether their expression levels were altered in response to the silencing of SlP4H3 (Supplementary Figure 1). The transcript abundance of seven P4Hs was detected, and among them SlP4H1, SLP4H2, and SlP4H5 exhibited similar to wild type expression levels (Supplementary Figure 1). However, only SlP4H9 showed significant upregulation in all three RNAi lines, while SlP4H4, SlP4H6, and SlP4H8 showed significant upregulation only in two and one RNAi lines, respectively (Supplementary Figure 1). No detectable expression of SlP4H7 was observed. These results indicate that no other tomato P4H was downregulated in the SlP4H3 RNAi lines.

The AZ of fully open flowers is comprised of six to eight layers of small, disk-shaped cells with dense cytoplasm, which is considered a very distinct, specialized structure of the pedicel (Tabuchi et al., 2001). This structure was investigated in the SlP4H3 RNAi lines by longitudinal sections of the AZ of fully open flowers indicating similar anatomical alterations in all three RNAi lines (Figure 2B). The RNAi AZ specialized cells were not positioned along a straight line as in the wild type but the AZ plane was curved as clearly shown by the outline of the dotted lines (Figure 2B). Moreover, a variation in the number of AZ cell layers was observed with lower number of cell layers in the central parenchymatic region (Figure 2B).

**Figure 4:**
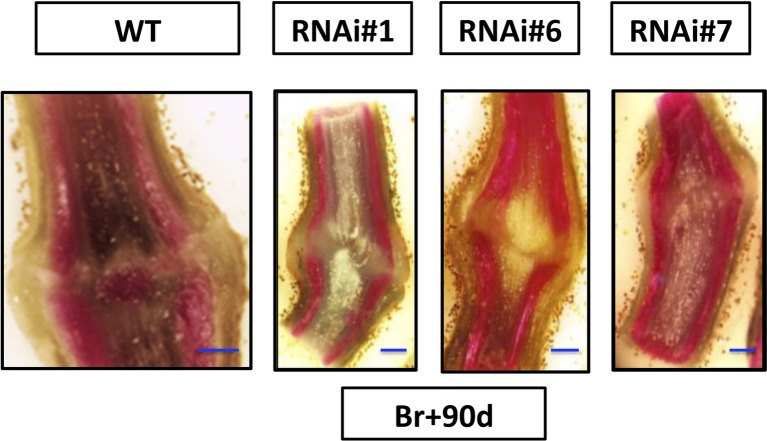
Analysis of longitudinal sections of fruit AZs 90 days after breaker. Fruit AZs 90 days after breaker were stained for lignin deposition with phloroglucinol-HCl in the three RNAi lines #1, #6, and #7 and wild type. The curved fracture plane can be observed in the AZs of the RNAi lines compared to the wild type (WT). The blue line represents 1 mm.

Moreover, lignin deposition was observed in the AZ of the wild type sections at 90 days after breaker while no lignification was detected in the AZs of the RNAi lines’ pedicel at 90 days after Breaker (Figure 4). These results might be related to the delay in abscission of the overripe fruits of SlP4H3 RNAi lines.

**Figure 5:**
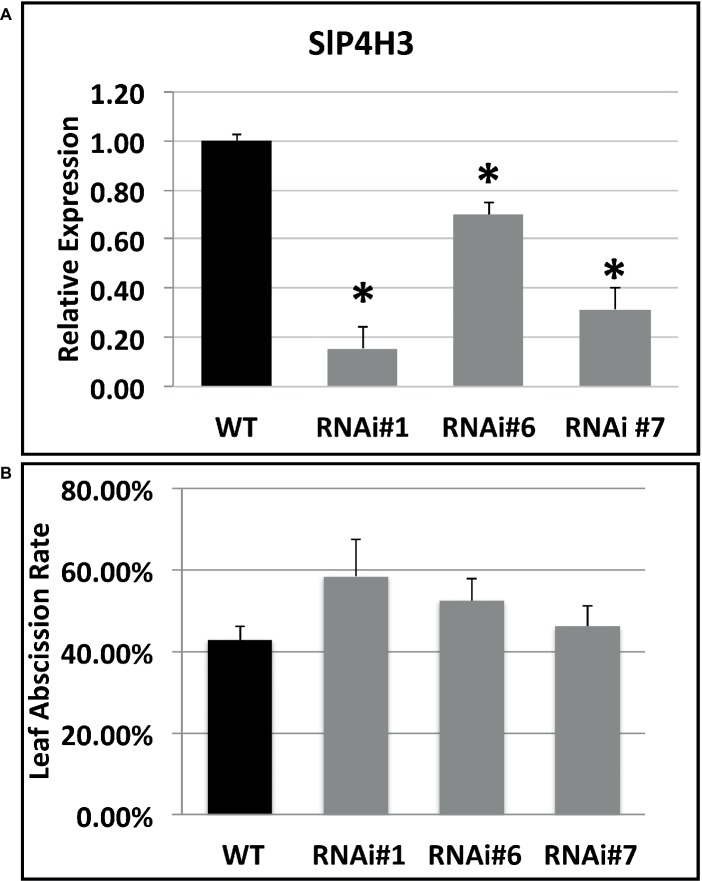
Expression analysis of the SlP4H3 in petiole AZs and leaf abscission rate after 48 h of ethylene treatment. **(A)** Relative expression of SlP4H3 transcript in three RNA lines and WT petiole AZs. The relative expression was calculated according to the comparative Ct method by using actin as internal standard. The asterisk indicates statistically significant differences. **(B)** The petiole abscission kinetics expressed as percentages of pedicel abscission were determined after deblading the leaf explants and treating them with approximately 15 μL L^−1^ of ethylene in the three SlP4H3 RNAi lines #1, #6, and #7 and the wild type. Three biological replicates were performed.

The SlP4H3 involvement in the progression of ethylene-induced petiole abscission was also investigated. Initially, the expression of SlP4H3 was determined in leaf AZ explants of the three RNAi lines and wild type (Figure 5A). Statistically significant downregulation was observed in all RNAi lines compared to wild type (Figure 5A). In addition, the leaf AZ explants were treated with 15 μl L^−1^ ethylene for 48 h in order to determine whether there are differences in the percentage of the abscission rate in response to ethylene. No statistically significant differences were observed among the three RNAi lines and wild type, indicating that ethylene-induced leaf abscission might not be affected by the levels of expression of SlP4H3 (Figure 5B).

### Gene Expression in Fruit Abscission Zones 90 Days After the Breaker Stage

**Figure 6:**
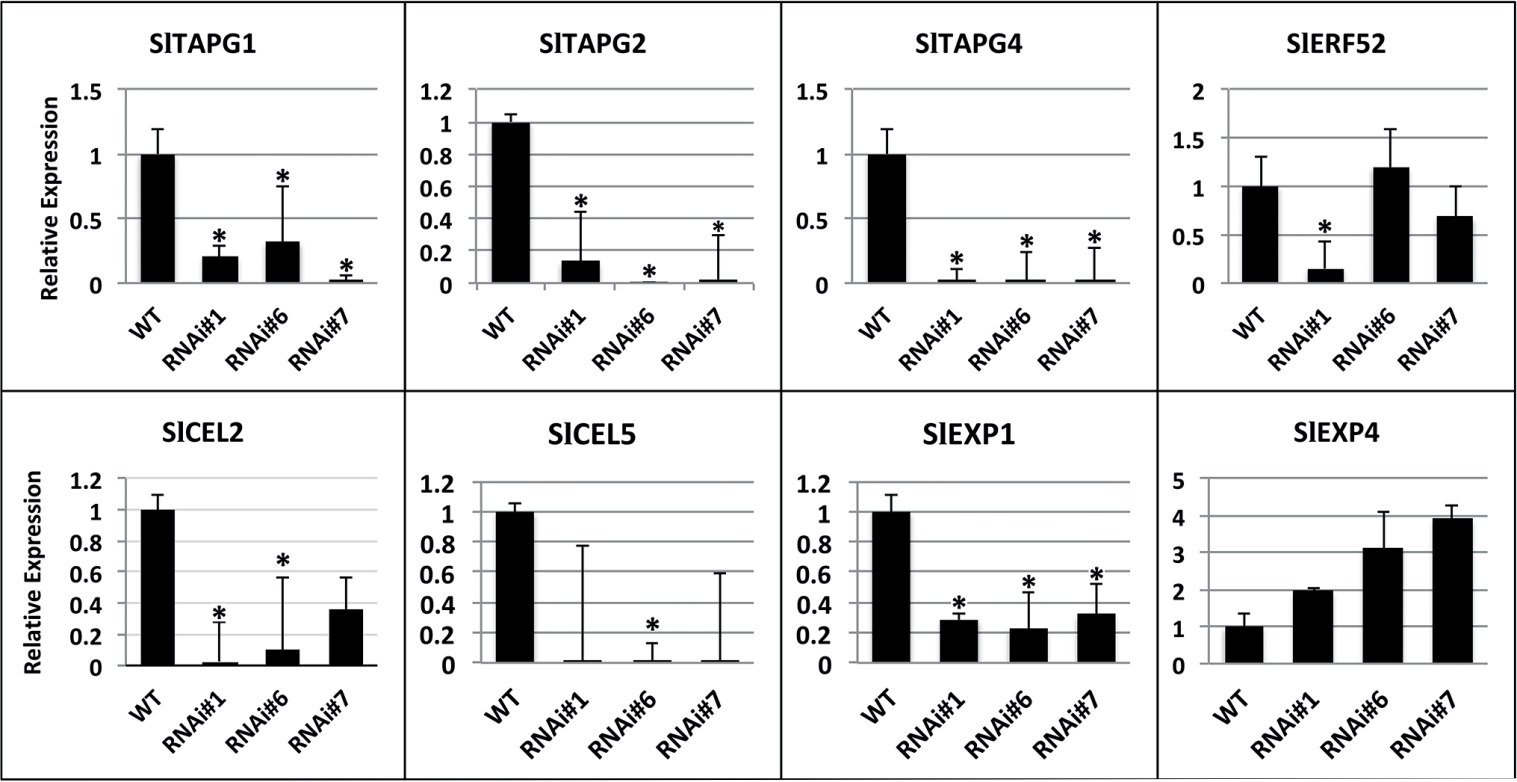
Expression analysis of SlTAPG1 (polygalacturonase), SlTAPG2, SlTAPG4, SlCEL2, (cellulase), SlCEL5, and SlEXP1 (expansin 1), SlEXP4, SlERF52 genes in AZs of SlP4H3 RNAi lines #1, #6, and #7 and wild type (WT). The relative expression was calculated according to the comparative Ct method by using actin as internal standard. The asterisk indicates statistically significant differences.

The expression of fruit abscission-specific genes was characterized in order to investigate the molecular basis of this delay in abscission. The patterns of expression of cell wall hydrolases such as SlTAPG1, SlTAPG2, SlTAPG4, and SlCEL2, SlCEL5 as well as additional mRNAs such as SlEXP 1, SlEXP4, and SlERF52 were determined (Figure 6). SlEXP1 is considered a fruit ripening-regulated expansin with high levels of expression in tomato leaf AZs while SlEXP4 showed similar levels of transcript abundance in both leaf and flower AZs (Sundaresan et al., 2016). In addition, SlEXP1 was also shown to be upregulated in flower AZs after 6 h of ethylene treatment (Wang et al., 2013). Total RNA was extracted from RNAi lines #1, #6, and #7 fruit AZs at 90 days after the breaker stage and wild type and used for qPCR analysis.

The transcript levels of the five cell wall hydrolases, SlTAPG1, SlTAPG2, SlTAPG4, and SlCEL2, SlCEL5, were determined in the overripe AZs considering the high levels of expression of SlTAPG1, SlTAPG2, and SlTAPG4 in tomato flower AZs (Kalaitzis et al., 1997; Sundaresan et al., 2016) and of SlCEL5 and SlCEL2 in both flower and leaf AZs (Sundaresan et al., 2016). The expression levels of SlTAPG1, SlTAPG2, SlTAPG4, and SlEXP1 were significantly downregulated by approximately 80–90% in all three SlP4H3 lines, justifying the delay in overripe fruit abscission compared to the wild type (Figure 6). However, the expression of SlCEL2 and SlCEL5 decreased in the RNAi lines but was not statistically significant for RNAi line #7 and RNAi lines #1 and #7, respectively (Figure 6). Moreover, the transcript levels of EXP4 were not altered significantly in the RNAi lines (Figure 6). The expression of SlERF52 was not altered significantly in RNAi lines #6 and #7, just downregulation in RNAi line #1 was observed (Figure 6).

**Figure 7:**
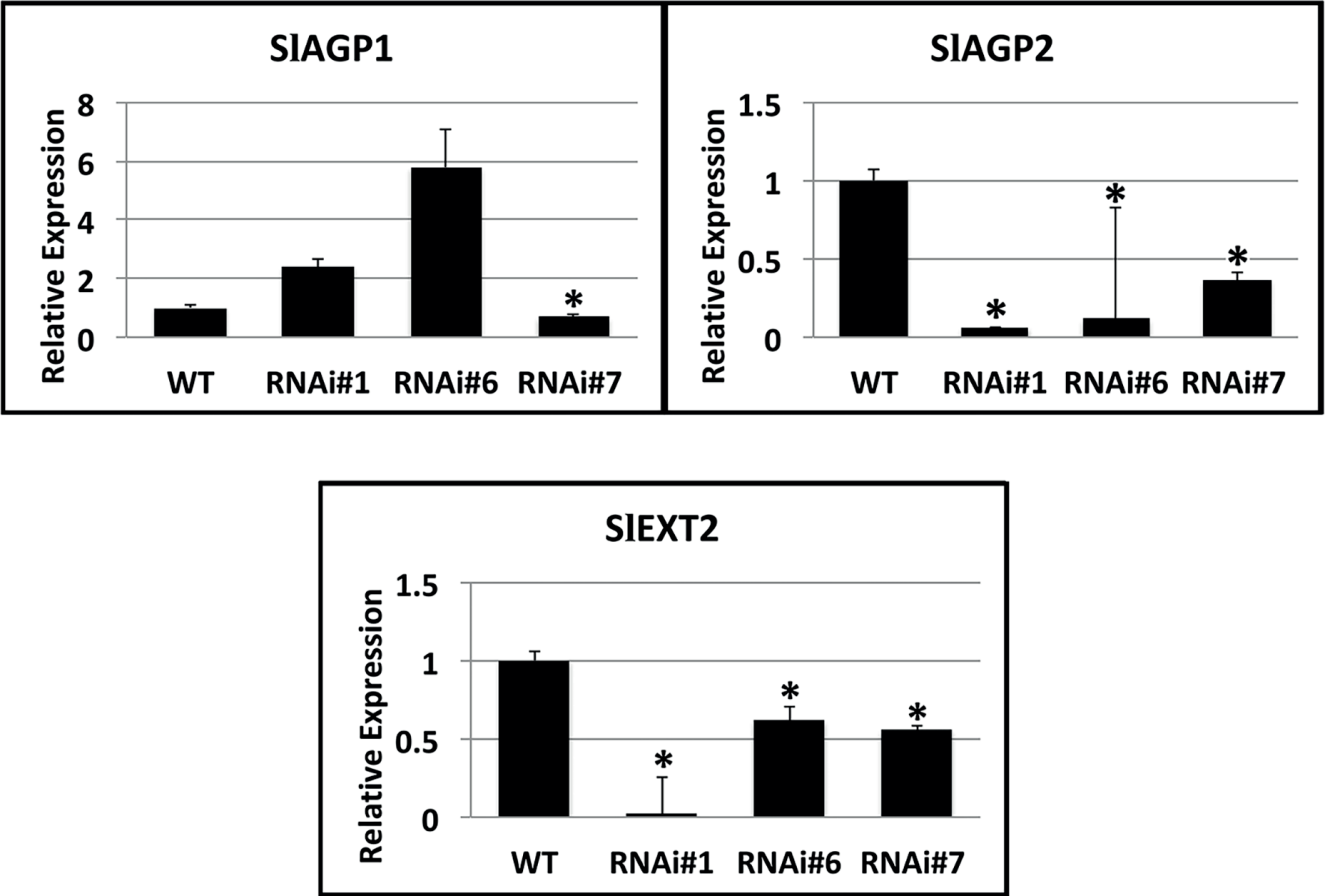
Expression analysis of SlAGP1 (arabinogalactan protein 1) (Solyc02g092790.2.1), SlAGP2 (Solyc07g053640.1), and SlEXT2 (extensin 2) (solyc09g098510.3) genes in AZs of SlP4H3 RNAi lines #1, #6, and #7 and wild type (WT). The relative expression was calculated according to the comparative Ct method by using actin as internal standard. The asterisk indicates statistically significant differences.

Considering that the substrate proteins for proline hydroxylation are AGPs and extensins, a search in the Affymetrix microarrays of SlP4H3 line #6 and wild type fruit AZs at 30 and 90 days after the breaker stage for transcripts encoding these polypeptides resulted in the identification of two SlAGPs, SlAGP1 and SlAGP2, and one extensin, SlEXT2 (Figure 7). RT-qPCR analysis indicated that SlAGP2 and SlEXT2 showed significantly lower levels of expression compared to wild type (Figure 7). The SlAGP1 transcript showed similar levels in RNAi lines #1 and #6 and lower levels in line #7 (Figure 7). These results indicate overall lower levels of SlAGPs and extensin transcript abundance in RNAi lines.

## Discussion

P4Hs catalyze the post-translational modification of proline hydroxylation in HRGPs such as AGPs and extensins as well as in hormone peptides such as the *Arabidopsis* IDA and CLAVATA3 (Matsubayashi, 2014).

With regard to AGPs, it was observed that Arabinogalactan was secreted in the AZs of *Arabidopsis* flowers after staining with the β-Yariv reagent which is known to bind AGPs (Stenvik et al., 2006). The presence of AGPs by using the JIM13 antibody was detected only in the IDA overexpressing flower AZs and not in wild type or IDA knock out mutant (Stenvik et al., 2006). This expression of the JIM13-bound epitopes was attributed to the upregulation of the AGP24 transcript in the IDA overexpressing floral AZs (Stenvik et al., 2006) indicating that AGPs might have a physiological role in progression of flower abscission. This is in accordance to our results that two SlAGPs and one extensin are expressed in the overripe fruit AZs. However, lower levels of SlAGP2 and extensin 2 were observed in their expression patterns in the RNAi lines compared to wild type, despite the fact that SlP4Hs catalyze a post-translational modification. These results indicate that proline hydroxylation might be involved in the regulation of transcription factors’ stability considering similar involvement in all metazoan species (Semenza, 2010). There are proline hydroxylation motifs which were identified in the polypeptide sequences of transcription factors induced by hypoxia in *Arabidopsis* (Vlad et al., 2007). In this case, alterations in the expression of SlP4Hs might induce alterations in the activity of transcription factors resulting in changes in gene expression of a wide array of genes comprising also AGPs and extensins as shown in Figure 7.

However, no differences were observed for the expression of SlAGP1 in lines #6 and #7. This suggests that there might be alterations for SlAGP1 at post-transcriptional level and probably at their protein content as previously reported in tomato (Fragkostefanakis et al., 2014). VIGS-induced suppression of three tomato P4Hs was accompanied by a decrease in protein content of AGPs and extensins in roots and shoots (Fragkostefanakis et al., 2014).

It was previously reported that RNAi-induced knock down of SlERF52 delayed pedicel abscission at the stage of anthesis and that the cause of this delay was attributed to the suppression of expression of SlTAPGs and cellulases (Nakano et al., 2014). In the SlP4H3 RNAi lines, the expression of the SlERF52 was not consistent showing minor alterations in expression levels compared to the wild type for lines #6 and #7 and lower expression levels for line #1 (Figure 6). These results suggest that SlERF52 might not be involved in the delay of overripe fruit abscission in the SlP4H3 RNAi lines considering that no significant changes in its expression were observed.

VIGS-induced suppression of SlTAPG1 expression probably resulted in the silencing of all five PGs in the petiole AZs and therefore in the inhibition of tomato petiole abscission (Jiang et al., 2008). In the same report, the silencing of two expansins and two endoglucanases did not affect flower abscission (Jiang et al., 2008). Therefore, the significant downregulation of SlTAPG1, SlTAPG2, and SlTAPG4 in the three SlP4H3 RNAi lines might be considered responsible for the delay in overripe fruit abscission of SlP4H3 suppression lines regardless of the levels of expression of endoglucanases and expansins (Figure 6). It is interesting to note that the regulation of a post-translational modification, such as proline hydroxylation, induces alterations in the expression levels of a plethora of genes with different functions. There is an example in which the stability of a transcriptional activator such as the hypoxia-inducible factor 1α (HIF-1α) which is considered a global regulator of hypoxic response in mammalian systems is regulated by proline hydroxylation (Koyasu et al., 2017). Three prolyl 4 hydroxylases (PHDs) trigger the ubiquitin-dependent degradation of HIF-1α and by this way regulate the stability of this transcription factor (Koyasu et al., 2017). However, no additional lines of evidence were reported up to now indicating proline hydroxylation of transcription factors in plants.

Moreover, a KNOTTED-LIKE HOMEOBOX (KD1) protein is also considered a regulator of tomato leaf and flower abscission (Ma et al., 2015). Decrease of KD1 mRNA levels resulted in significant retardation of petiole and pedicel abscission while overexpression of this gene in a semidominant mutant, *Petroselinum*, accelerated abscission (Ma et al., 2015). Although suppression of SlP4H3 transcript caused a delay of overripe fruit abscission as in KD1-silenced tomato plants, preliminary analysis of transgenic lines overexpressing SlP4H3 did not show any acceleration in overripe fruit abscission (data not shown). Moreover, the suppression of SlP4H3 delayed pedicel abscission but did not affect petiole abscission indicating that proline hydroxylation might be involved in a different abscission regulatory mechanism compared to KD1 or alternatively, other P4H(s) might be involved in the regulation of leaf abscission in tomato. These results suggest that there might be differences between petiole and pedicel abscission at the molecular level in tomato, although global transcriptome analysis indicated similar regulation of flower and leaf abscission (Sundaresan et al., 2016). However, it should be noted that P4Hs catalyze a post-translational modification which is not related to transcriptome regulation.

Antibodies for immunolocalization of xyloglycan endotransglycosylase/hydrolases (XTH) and expansins in tomato flower and fruit AZs showed detection of both proteins in flower AZ but complete lack of expression in overripe fruit AZ (Tsuchiya et al., 2015). Intense labeling was observed only in the vascular bundles of overripe fruit pedicels (Tsuchiya et al., 2015). Expansin 1 (SlEXP1) was strongly expressed in AZs 90 days after breaker (Figure 6). Although these results are not in accordance with the report by Tsuchiya et al. (2015), it should be taken into consideration the different fruit developmental stages which were used for the immunolocalization of XTH and EXP and their gene expression patterns by qPCR analysis, respectively. The overripe fruit AZs used for the immunolocalization studies probably refer to fruit ripening stages just several days after the red ripe stage (Tsuchiya et al., 2015).

Significant structural differences were observed in the AZ of the RNAi lines compared to the wild type considering that the fracture plane was curved and not across a line (Tabuchi et al., 2001), indicating alterations in the structure and development of pedicel AZ. The tomato pedicel AZ is comprised of cells arrested in an undifferentiated stage (van Nocker, 2009) and the observed curved fracture plane suggests that the developmental program of differentiation of specific cells was not executed properly. Therefore, the expression of the genes involved in the development of the pedicel AZ in RNAi lines needs to be determined at the early stage of flower AZ development (Ito and Nakano, 2015).

Moreover, extensive lignification was observed in the AZs of the overripe but not of mature green fruits (Tsuchiya et al., 2015). Similar lignin deposition was also detected in the AZs 90 days after breaker only in the wild type but not in the RNAi lines (Figure 4). This lignification was associated with abscission-specific activation (Tsuchiya et al., 2015) which is in accordance with the lack of lignification in the AZ of the RNAi lines 90 days after breaker considering the delay in abscission activation. According to the previous (Iwai et al., 2013; Tsuchiya et al., 2015) and this report, lignin deposition might be considered a marker of abscission activation. Strong lignin deposition was also observed in the vascular bundles of AZs at 90 days after breaker in the wild type and RNAi lines. Similar results were also reported in previous studies (Iwai et al., 2013; Tsuchiya et al., 2015).

The suppression of SlP4H3 expression resulted in the delay of the overripe tomato fruit abscission due to the significant downregulation of the abscission polygalacturonases and cellulases as well as an expansin. Moreover, alterations were also observed in the structure of the AZs the significance of which remains to be elucidated. These results suggest that proline hydroxylation might play a key role in the induction of abscission cell wall hydrolases indicating an additional level of regulation of tomato pedicel abscission. Therefore, further studies are under way to investigate the AZ of red ripe fruits in response to ethylene in the RNAi as well as overexpression lines.

## Author Contributions

PK conceived and designed the work. PK, AP, CB, SA, AP, AK, KM, DD, KB, and DK were involved in the experiments, analysis, and interpretation of the data. PK, AP, CB, SA, AP, AK, KM, DD, KB, and DK were involved in drafting the work. All authors revised and approved the final version.

### Conflict of Interest Statement

The authors declare that the research was conducted in the absence of any commercial or financial relationships that could be construed as a potential conflict of interest.

## Footnotes

^1^http://tomexpress.toulouse.inra.fr/

^2^http://ted.bti.cornell.edu/

## Supplementary Material

SUPPLEMENTARY FIGURE 1 Expression analysis of SlP4H1 (solyc02g064750), SlP4H2 (Solyc06g054490), SlP4H4 (Solyc02g067530), SlP4H5 (Solyc01g080530), SlP4H6 (Solyc02g087490), SlP4H8 (Solyc03g033320), SlP4H9 (Solyc11g005200) genes in AZs of SlP4H3 RNAi line #1, #6 and #7 and wild type (WT). The relative expression was calculated according to the comparative Ct method by using actin as internal standard. The asterisk indicates statistically significant differences.

SUPPLEMENTARY TABLE 1 Table of primers used for the qPCR analysis, the subcloning of SlP4H3 cDNA in GATEWAY vector, and amplification of NPTII partial cDNA fragment.
