# Supplementary figures and images for: Suppression of a Prolyl 4 Hydroxylase Results in Delayed Abscission of Overripe Tomato Fruits
Source: Front Plant Sci. 2019 Mar 28;10:348. doi: 10.3389/fpls.2019.00348 (PMC6447859; doi:10.3389/fpls.2019.00348)

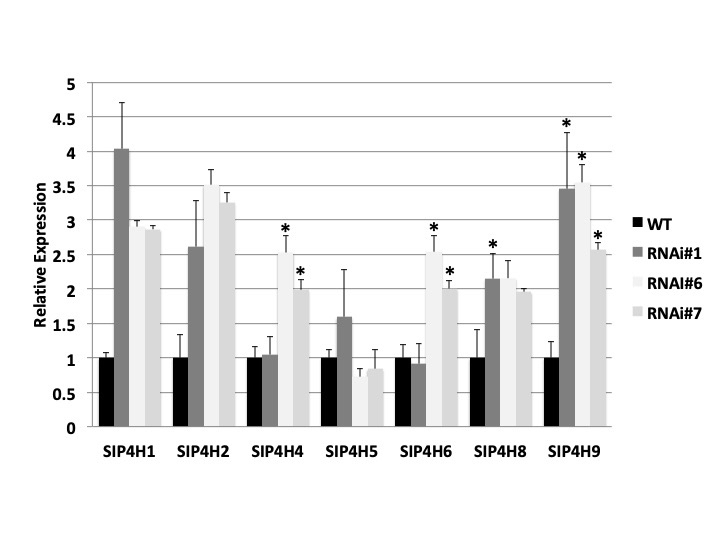

Supplement: SUPPLEMENTARY FIGURE 1 — Expression analysis of SlP4H1 (solyc02g064750), SlP4H2 (Solyc06g054490), SlP4H4 (Solyc02g067530), SlP4H5 (Solyc01g080530), SlP4H6 (Solyc02g087490), SlP4H8 (Solyc03g033320), SlP4H9 (Solyc11g005200) genes in AZs of SlP4H3 RNAi line #1, #6 and #7 and wild type (WT). The relative expression was calculated according to the comparative Ct method by using actin as internal standard. The asterisk indicates statistically significant differences. [file Image_1.JPEG]
