# Supplementary material for: Suppression of a Prolyl 4 Hydroxylase Results in Delayed Abscission of Overripe Tomato Fruits
Source: Front Plant Sci. 2019 Mar 28;10:348. doi: 10.3389/fpls.2019.00348 (PMC6447859; doi:10.3389/fpls.2019.00348)
Supplement: SUPPLEMENTARY TABLE 1 — Table of primers used for the qPCR analysis, the subcloning of SlP4H3 cDNA in GATEWAY vector, and amplification of NPTII partial cDNA fragment. [file Table_1.DOCX]

**Supplementary Table 1.** Table of primers used for the qPCR analysis, the subcloning of SlP4H3 cDNA in GATEWAY vector, and amplification of NPTII partial cDNA fragment.

| **Gene** | **Primer name** | **Sequence (5' - 3')** |
| --- | --- | --- |
| *SlP4H3* | SlP4H3-attb1  SlP4H3-attb2 | GGG GAC AAG TTT GTA CAA AAA AGC AGG CTA ATG GCA GTG AAA GGA AG  GGG GAC CAC TTT GTA CAA GAA AGC TGG GTC TTA TCA CGC CCT CTT |
| NPTII | PnptII-F  PnptII-R | GAGGCAGCGCGGCTATC  GCGGTCCGCCACACCCA |
| *SlActin* | SlActin_F SlActin_R | GTCCCTATTTACGAGGGTTATGCT GTT CAG GTTCAGCAGTGGTGGTGAACA |
| *SlAGP1* | SlAGP1_F  SlAGP1_R | GCACCTTCTCCCGATATGAT  CCCATCCAGCTACCAAACTT |
| *SlAGP2* | SlAGP2_F  SlAGP2_R | GCAAGGATTAAGCGACACAA  GAAAATCGCGTCTCACTGAA |
| *SlCEL5* | SlCEL5_F  SlCEL5_R | ﻿GGGATGACAAGAGACCTGGAA  GCCTGGAATCAATGAGCAGA |
| *SlEXT2* | SlEXT2_F  SlEXT2_R | GTTCTCCGCCACCACCAGTC  GAAGCAGAAGCAAAGTGGTCC |
| *SlTAPG1* | SlTAPG1_F  SlTAPG1_R | GCTCGGGTATAAAGATAAGTGATGTG  TGTTATGCCGCTACATGGATTAC |
| *SlTAPG4* | SlTAPG4_F  SlTAPG4_R | GTGCTAGTCTTTGGGCTTGC  TCATCGCGTTATGACAACCA |
| *SlEXP1* | SlEXP1_F  SlEXP_R | ATTACGCGTTGCCAAATGACA  AGCGCGGTACTGAGCAAGTT |
| *SlP4H3* | SlP4H3_F  SlP4H3_R | GAATAGAGCCACCACACATGAA  CGAATGAAGCATCTCACATCTT |
| *SlEXP4* | SlExp4_F  SlExp4_R | GCAAATGGTGTCTTCCTGGT  GCACGGTACAGTCCGATTTT |
| *SlERF52* | SlERF52_F  SlERF52_R | CCATGTCTCGACCACAACAA  CATAGGTTCCCAGCCATATCC |
| Sl*TAPG2* | SlTAPG2_F  SlTAPG2_R | GGGTTCTGGTATAAAGATAAGCGATA  TCAAGTGTTATGCCGCTGCAC |
| *SlCel2* | SlCel2_F  SlCel2_R | TTTTCACCAAACCCAAACCCA  TCTTGAAAATAGGGTCTGGCGTCT |
| *SlP4H1* | SlP4H1_F  SlP4H1_R | GGTTTGAAAGTGAAGCCACGC CAATTGTACCATTTGGAAGCAGTGA |
| *SlP4H2* | SlP4H2_F  SlP4H2_R | CGAGAGTCGAGACAGTGACAGTAA  GCATGCTTGCACCTGTATGTGGA |
| *SlP4H4* | SlP4H4_F  SlP4H4_R | CAATGGACCGTCAACTTCCTT  CTACCAGTCACCAATTTCAGCA |
| *SlP4H5* | SlP4H5_F  SlP4H5_R | TCCCCGAAGCAGAGGTATCA CCTCTTTGCACACTCGGACA |
| *SlP4H6* | SlP4H6_F  SlP4H6_R | CAAGGAAGAATGTGAATACTTGATAA  GTACGAACCCTGCTATCTTTACT |
| *SlP4H8* | SlP4H8RT_F  SlP4H8RT_R | CAGCTTTCAACCTCAAGCC  GTGCCGAGTGGATTTACTTTAC |
| *SlP4H9* | SlP4H9RT_F  SlP4H9RT_R | CTATGGCTGGCACTGGTAAAT  ACAGTTCTTTGCCTCATCCA |
